# Raman and FT-IR Spectroscopy Coupled with Machine Learning for the Discrimination of Different Vegetable Crop Seed Varieties

**DOI:** 10.3390/plants14091304

**Published:** 2025-04-25

**Authors:** Stefan M. Kolašinac, Marko Mladenović, Ilinka Pećinar, Ivan Šoštarić, Viktor Nedović, Vladimir Miladinović, Zora P. Dajić Stevanović

**Affiliations:** 1 Department of Agrobotany, Faculty of Agriculture, University of Belgrade, Nemanjina 6, 11180 Belgrade, Serbia; ilinka@agrif.bg.ac.rs (I.P.); sostaric@agrif.bg.ac.rs (I.Š.); dajic@agrif.bg.ac.rs (Z.P.D.S.); 2 Plant Breeding Department, Maize Research Institute Zemun Polje, Slobodana Bajića 1, 11185 Belgrade, Serbia; mmladenovic@mrizp.rs; 3 Department of Food Technology and Biochemistry, Faculty of Agriculture, University of Belgrade, Nemanjina 6, 11130 Belgrade, Serbia; vnedovic@agrif.bg.ac.rs; 4 Institute of Soil Science, Teodora Drajzera 7, 11000 Belgrade, Serbia; vladimir.miladinovic33@gmail.com

**Keywords:** merging spectra, vibrational spectroscopy, support vector machine (SVM), vegetable seed, breeding

## Abstract

The aim of this research is to investigate the potential of Raman and FT-IR spectroscopy as well as mathematical linear and non-linear models as a tool for the discrimination of different seed varieties of paprika, tomato, and lettuce species. After visual inspection of spectra, pre-processing was applied in the following combinations: (1) smoothing + linear baseline correction + unit vector normalization; (2) smoothing + linear baseline correction + unit vector normalization + full multiplicative scatter correction; (3) smoothing + baseline correction + unit vector normalization + second-order derivative. Pre-processing was followed by Principal Component Analysis (PCA), and several classification methods were applied after that: the Support Vector Machines (SVM) algorithm, Partial Least Square Discriminant Analysis (PLS-DA), and Principal Component Analysis-Quadratic Discriminant Analysis (PCA-QDA). SVM showed the best classification power in both Raman (100.00, 99.37, and 92.71% for lettuce, paprika, and tomato varieties, respectively) and FT-IR spectroscopy (99.37, 92.50, and 97.50% for lettuce, paprika, and tomato varieties, respectively). Moreover, our novel approach of merging Raman and FT-IR spectra significantly contributed to the accuracy of some models, giving results of 100.00, 100.00, and 95.00% for lettuce, tomato, and paprika varieties, respectively. Our results indicate that Raman and FT-IR spectroscopy coupled with machine learning could be a promising tool for the rapid and rational evaluation and management of genetic resources in ex situ and in situ seed collections.

## 1. Introduction

Modern field and vegetable crop production are highly dependent on the certified varietal seed, the production, processing, and packing of which are officially controlled according to a set of legal harmonized procedures [1]. Inter alia, certified varietal seed must fulfill certain quality conditions prescribed by law in order to be placed on the market where it is available to farmers. In addition to germination percentage, varietal identity and genetic purity are the most important characteristics of seed quality [2]. The varietal identification and genetic purity monitoring of plant varieties’ seeds enable the protection of the developer’s intellectual property rights, the plant breeders’ rights, which are guaranteed by the UPOV Convention [3], as well as the rights of all participants in crop production. Therefore, the unambiguous identification of varieties is important for the prevention of unauthorized commercial use, misuse, and forgery [4]. On the other hand, genetic purity monitoring of varietal seed is essential because a high level of genetic purity must be achieved to ensure that improvement in agronomic performance created by breeders will be delivered to the farmers, food technology, and, at the very end, to the consumers [5].

Standard protocols for screening varietal seed’s genetic purity and identity, standardized by the International Rules for Seed Testing (ISTA Rules), are mainly based on the isozyme (protein) analysis–electrophoresis methods (e.g., ultrathin-layer isoelectric focusing–UTLIEF) [6] and on the field grow-out test, which uses UPOV (International Union for the Protection of New Varieties of Plants) morphological descriptors. The grow-out test, although time-consuming, partially subjective, limitedly discriminatory, expensive, and environment-sensitive, is still the most commonly used method for genetic purity testing [7]. However, the grow-out test, as a traditional method based on morphological markers, cannot provide sufficient information about the range of specific genetic attributes [5]. On the other hand, biochemical (isozymes) assays based on ultrathin-layer isoelectric focusing electrophoresis (UTLIEF) can clearly distinguish between varieties of many plant species, which has been reported by many researchers (e.g., [8,9,10]). Nevertheless, the biochemical markers (isozymes), although much more informative than the morphological ones, still exhibit considerable disadvantages, including environmental sensitivity, ability to screen only a small fraction of the genetic variability, and, sometimes, insufficient discriminative performances [9].

It is well established that PCR (Polymerase Chain Reaction)-based DNA molecular markers provide more discriminative varietal identification than biochemical and morphological markers. Many experiments with different PCR-DNA markers used for varietal identification and purity testing have been performed (e.g., [11,12,13,14,15]). All such studies confirmed that the DNA markers are an extremely effective tool for genetic purity assessment. However, due to high analytical costs, complex experimental procedures, and a lack of automation, DNA markers have not significantly replaced traditional grow-out tests and biochemical assays for varietal identification and genetic purity, especially in the case of large-scale and commercial seed quality testing [9]. All methods used in varietal identification and purity testing have certain limitations, including sample preparation processes, which are mostly destructive and time-consuming.

So far, several non-destructive instrumental methods have been applied for testing genetic purity on the seed material of different crops, such as FT-NIR (Fourier transform near-infrared) spectroscopy [16], FT-IR spectroscopy [17], NIR [18], and Raman spectroscopy [19,20], and seed authenticity by NMR [21,22,23,24] and mass spectrometry [25,26]. Raman scattering (RS) and Fourier Transform Infrared (FT-IR) absorption are vibrational spectroscopy techniques widely used for label-free, non-invasive, non-preparatory, and low-cost optical analysis in various industrial and scientific fields [27]. RS and FT-IR are complementary techniques for the structural analysis of any molecule where the RS is active for anti-symmetric vibrations that change the dipole moment, while the FT-IR is active for symmetric vibrations that change the polarizability [28]. IR absorption spectroscopy, known for sensitivity to polar bonds such as O–H or N–H, is commonly used to identify the functional groups of different compounds, whereas Raman spectroscopy, sensitive to bonds such as C=C, S–S, or C–S4, is mostly used to identify skeletal molecular structures.

The aim of this research was to investigate the potential of Raman and FT-IR spectroscopy combined with different machine learning chemometric models in the discrimination of different crop varieties within three widely grown vegetable species: paprika (*Capsicum anuum* L.), tomato (*Lycopersicon esculentum* Mill), and lettuce (*Lactuca sativa* L.). The microspectroscopy-based chemometrics was performed to find the best possible models for seed discrimination within the species, i.e., a target crop (reflecting varietal variability). The models were not performed among vegetable crops (different species), especially because of the fact that their seeds differ macroscopically, i.e., in size, shape, color, etc. Thus, there was no reason to assess the interspecific differences by vibrational spectroscopy techniques. To the best of our knowledge, this is the first attempt at applying vibrational spectroscopy (FT-IR and Raman) in the discrimination of seed material of selected vegetables.

## 2. Results

Spectra obtained by Raman and FT-IR spectroscopy were used for the development of the most appropriate chemometric models in the classification of seed varieties.

### 2.1. Raman Spectroscopy Seed Fingerprinting

**Figure 1:**
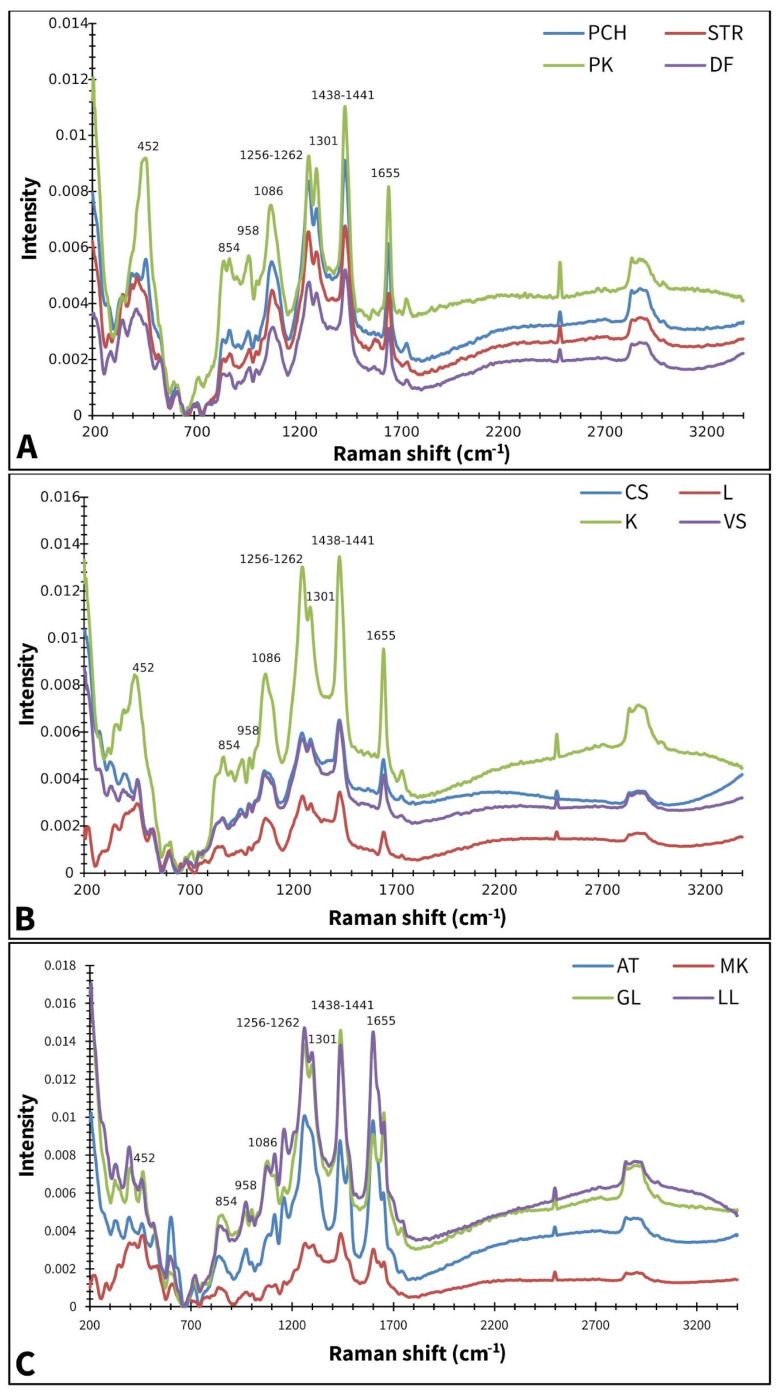
Baseline corrected and smoothed Raman spectra of paprika (**A**), tomato (**B**), and lettuce (**C**); AT—Atrakcija, MK—Majska kraljica, GL—Great Lake, LL—Ljubljanska ledenka, PCH—Palanačko čudo, STR—Strižanka, PK—Palanačka kapija, DF—Delfina, CS—Crvena Stena, L—Lider F1, K—King F1, VS—Volovsko srce.

Figure 1 displays the averaged Raman spectra of different paprika, tomato, and lettuce varieties. According to Seidler-Lozykowska et al. [29], bands around ~1655 cm^−1^ can be assigned to *ν*(C=C), the stretching vibration of unsaturated fatty acids and lignin. It is well known that lignin represents the main component of cell walls present in the seed coat [30]. Bands at ~1438–1441 cm^−1^ are characteristic of *δ*(CH_2_) scissoring deformation vibration, which can be assigned to lignins and lipids [31]. Bands at ~1301 cm^−1^ and 1256–1262 cm^−1^ are attributed to in-phase *δ*(=CH) methylene twisting and symmetric rocking vibration, respectively [32]. Medium-intensity bands are positioned at 1086 cm^−1^, involving the vibration of ν(C-O-C) glycosidic bonds [33]. While bands at 854 cm^−1^ were indicated on the C-O-C skeletal mode of α-anomers at 452 and 958 cm^−1^, and indicated on δ(CCH), δ(COH) of polygalacturonic (pectin) acid [34].

### 2.2. FT-IR Spectroscopy Seed Fingerprinting

**Figure 2:**
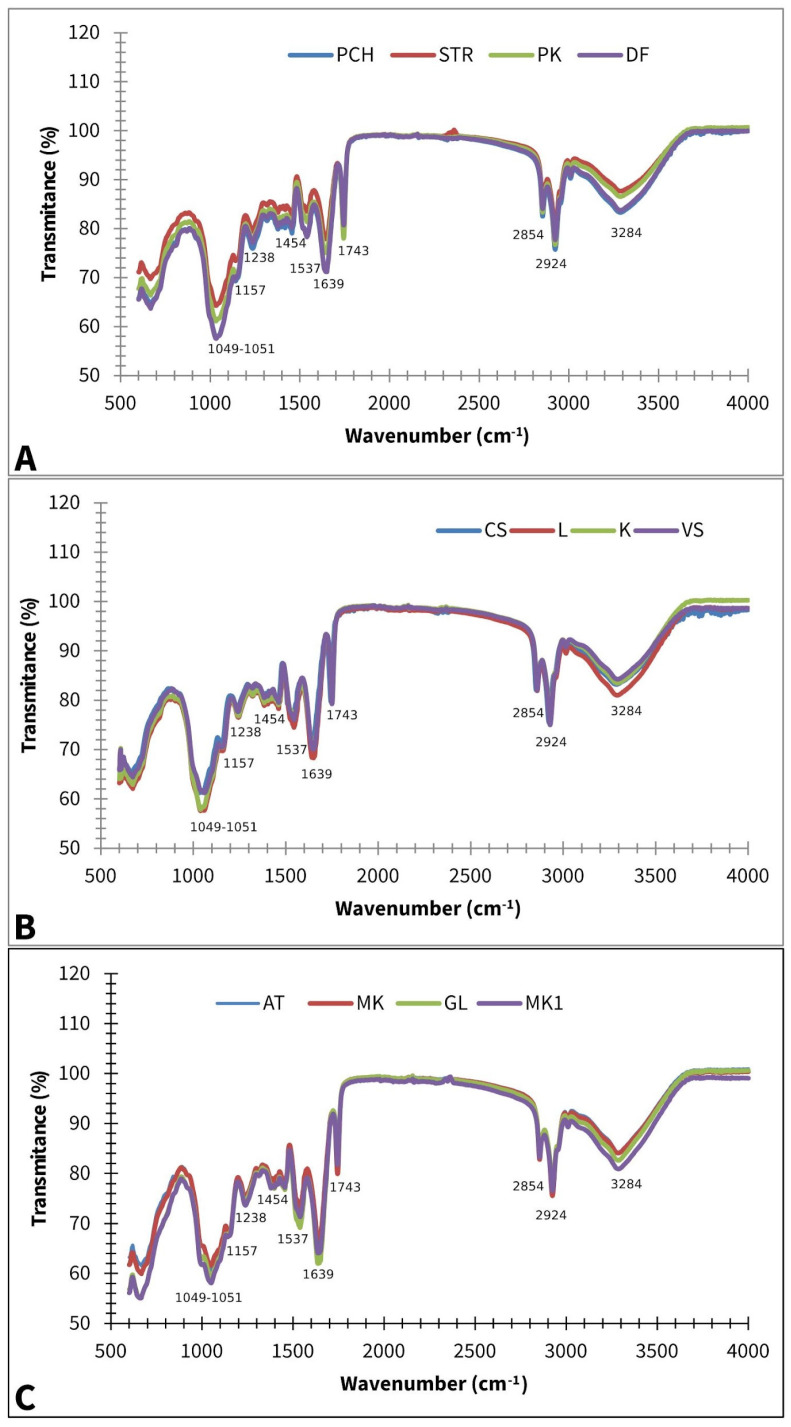
Baseline corrected and smoothed FT-IR spectra of paprika (**A**), tomato (**B**), and lettuce (**C**); AT—Atrakcija, MK—Majska kraljica, GL—Great Lake, LL—Ljubljanska ledenka, PCH—Palanačko čudo, STR—Strižanka, PK—Palanačka kapija, DF—Delfina, CS—Crvena Stena, L—Lider F1, K—King F1, VS—Volovsko srce.

Figure 2 displays averaged FT-IR spectra of different paprika, tomato, and lettuce varieties.

A strong band at 3284 cm^−1^ can be assigned to the OH stretching vibration, while a band at 3008 cm^−1^ could appear due to the =C-H *cis* stretching vibration, which can be connected with the presence of unsaturated lipids. Bands at ~2924 and ~2854 are asymmetric and symmetric stretching vibrations of CH_2_ groups, which can be associated with the hydrocarbon chains of the lipids or lignins [35]. The absorption at ~1743 cm^−1^ can be assigned to –C=O stretching, which is probably due to the presence of fatty acids or pectin [36]. The presence of a protein/Amide I structure is confirmed by 1639 cm^−1^, which can be associated with C=O and C-N stretching. The band at ~1537 cm^−1^ is due to N-H bending, which can be linked with Protein/Amide II [37]. The band at ~1454 cm^−1^ could be assigned to CH_2_ bending scissoring and is probably associated with lignin [38], which is a known constituent of many seed chemical profiles [39]. According to Alcantara et al. (2010) [40], the band at ~1238 cm^−1^ contributes to –C-O and -CH_3_ bending, probably because of fatty acid molecules, while the one at ~1157 cm^−1^ is related to -C-O stretching and -CH_2_- bending, which overlaps with the C-N stretching band of proteins. Phospholipids PO_2_ and P-O-C were also identified due to the absorption at ~1049–1151 cm^−1^ [40].

### 2.3. Chemometric Analysis of the Raman and FT-IR Spectra of Lettuce, Paprika, and Tomato Varieties

Because it is rather difficult to choose a pre-processing technique suitable for all spectra, several methods were applied. In order to enhance the signal-to-noise ratio and eliminate features and variations that are not part of the sample, UNV and a second-order Savitzky-Golay derivative were performed; Raman and FT-IR spectra preprocessed by these algorithms served as an input for Principal Component Analysis (PCA). PCA reduces dimensionality (number of variables) by creating new uncorrelated variables that successively maximize variance, called principal components (PCs). Further, PCA prevents the risk of building a too optimistic model (when the number of variables exceeds the number of samples).

**Figure 3:**
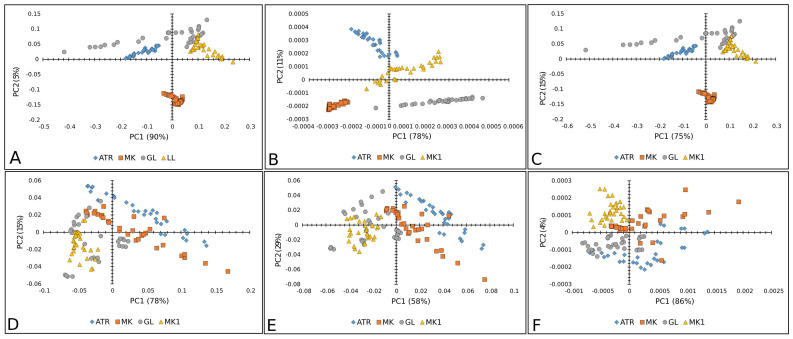
PCA score plots (PC1 vs. PC2) of lettuce varieties: Raman spectra combined with (**A**) BC+N, (**B**) BC+N+MSC, and (**C**) BC+N+MSC+2nd OD; FT-IR spectra with (**D**) BC+N, (**E**) BC+N+MSC, (**F**) BC+N+MSC+2nd OD. AT—Atrakcija, MK—Majska kraljica, GL—Great Lake, LL—Ljubljanska ledenka.

**Figure 4:**
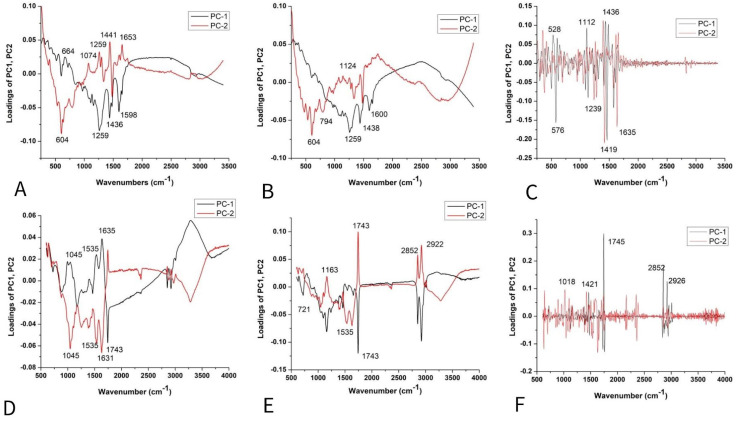
PCA loading plots of lettuce varieties: Raman spectra combined with (**A**) BC+N, (**B**) BC+N+MSC, and (**C**) BC+N+MSC+2nd OD; FT-IR spectra with (**D**) BC+N, (**E**) BC+N+MSC, (**F**) BC+N+MSC+2nd OD.

**Figure 5:**
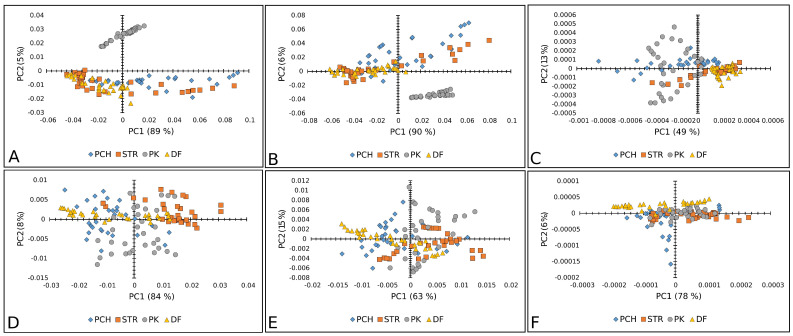
PCA score plots (PC1 vs. PC2) of paprika seed varieties: Raman spectra combined with (**A**) BC+N, (**B**) BC+N+MSC, and (**C**) BC+N+MSC+2nd OD; FT-IR spectra with (**D**) BC+N, (**E**) BC+N+MSC, (**F**) BC+N+MSC+2nd OD. PCH—Palanačko čudo, STR—Strižanka, PK—Palanačka kapija, DF—Delfina.

Figure 3, Figure 4 and Figure 5 show the results of the PC analysis of the Raman and FT-IR spectra of lettuce, paprika, and tomato varieties. In the case of the lettuce varieties, the PCA score plot of Raman spectra reveals a tendency toward a cluster formation in the investigated lettuce varieties. Accordingly, MK is well separated from the others, according to PC, while AT and LL are discriminated along PC1. This statement is true for all preprocessed algorithms used, but the clearest separation is obtained when SM+BC+UN+MSC is applied (Figure 3A–C). However, in the case of FT-IR spectra, it did not reveal any tendency toward a cluster formation because the analyzed data showed a correlation (Figure 3D–F).

The loading plot of PC2 (Figure 4A–C) shows the positive loadings responsible for the separation between the GL, LL, and ATR varieties from the MK. Examination of the PC2 shows many medium-positive contributions at 1074, 1259, ~1441, and 1653 cm^−1^, which could be attributed to unsaturated fatty acids and lignin [29,31,32].

The negative loading on PC2 (Figure 4A,B) indicated that proteins have a crucial effect on the differences of MK from other varieties [41]. According to the score plot based on the FT-IR spectra (Figure 4D–F) of lettuce seeds, it mainly indicated the similarities in chemical composition between varieties AT, MK, GL, and LL. The bands positioned at 1045, 1535, 1631, and 1743 cm^−1^ indicated similarities probably coming from carbohydrates, Amide I and II groups, and fatty acids [37], as well as bands at 2852 and ~2922 cm^−1^ involving the C=O stretching band of the fatty acids [38].

Figure 5A–C displays good separation when Raman spectra are used only for the PK variety of paprika, except when the second-order derivative was used as a preprocessing algorithm. Whereas the other three paprika varieties were mixed. In the case of FT-IR spectra, there was no separation between paprika varieties (Figure 5D–F).

**Figure 6:**
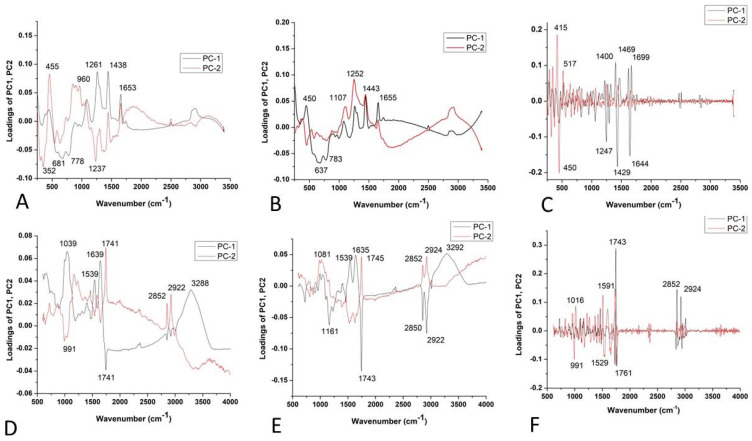
PCA loading plots of paprika seed varieties: Raman spectra combined with (**A**) BC+N, (**B**) BC+N+MSC, and (**C**) BC+N+MSC+2nd OD; FT-IR spectra with (**D**) BC+N, (**E**) BC+N+MSC, (**F**) BC+N+MSC+2nd OD.

The score plot (Figure 6A,B) suggests the existence of two groups of objects along the PC1 and PC2 axes; PK differs from other varieties. The loading plot shows that the variables with the highest positive contribution along the PC1 axis corresponded to the signals at 1261, 1438, and 1653 cm^−1^ (Figure 6A,B), attributed to unsaturated fatty acids and lignin [39,42,43]. Figure 6C indicates the similarities between the varieties based on unsaturated fatty acids and phenols [44], according to bands in the region from 1240 to 1700 cm^−1^ (Figure 6C). According to the score plot based on the FTIR spectra of paprika seeds, it indicates the similarities in chemical composition between varieties. The similar chemical composition is mainly based on fatty acids, Amide I and II groups, and probably carbohydrates (around 1740 cm^−1^, then 1638 and 1539 cm^−1^, and bands up to 1170 cm^−1^, respectively) [37] (Figure 6C–E).

**Figure 7:**
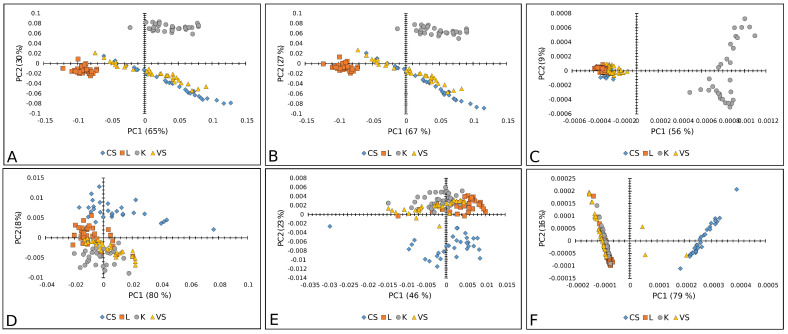
PCA score plots (PC1 vs. PC2) of tomato varieties: Raman spectra combined with (**A**) BC+N, (**B**) BC+N+MSC, and (**C**) BC+N+MSC+2nd OD; FT-IR spectra with (**D**) BC+N, (**E**) BC+N+MSC, (**F**) BC+N+MSC+2nd OD. CS—Crvena Stena, L—Lider F1, K—King F1, VS—Volovsko srce.

Furthermore, Figure 7 represents a PCA score plot of tomato Raman and FT-IR spectral data. The PK variety was visibly separated in the case of Raman spectra, while the CS variety was well separated by FT-IR spectra.

**Figure 8:**
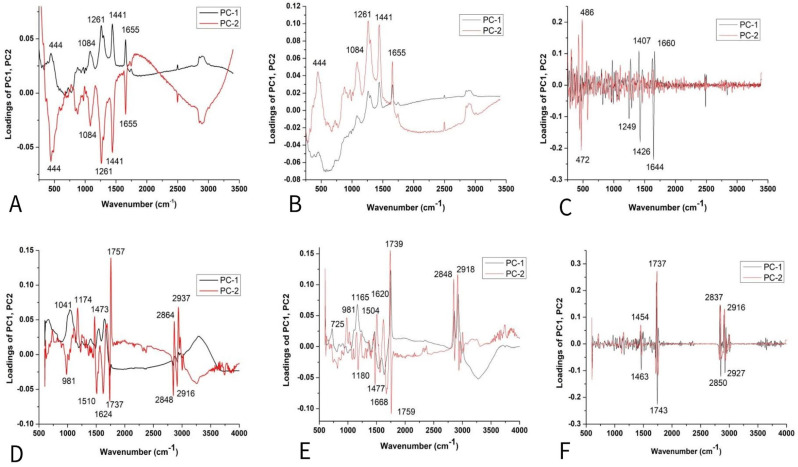
PCA loading plots of tomato seed varieties: Raman spectra combined with (**A**) BC+N, (**B**) BC+N+MSC, and (**C**) BC+N+MSC+2nd OD; FT-IR spectra with (**D**) BC+N, (**E**) BC+N+MSC, (**F**) BC+N+MSC+2nd OD.

The loading plots (Figure 5A,B) show that the variables with the highest positive contribution along the PC1 axis corresponded to the signals at 1261, 1441, 1655 cm^−1^, and 1084, 444 cm^−1^. Loadings indicated that K mainly differs from all other tomato varieties in unsaturated fatty acids, lignin, and polysaccharides [29,31,32,33].On the clear differences between mentioned clusters (Figure 8C), it mainly included bands indicated by unsaturated fatty acids assigned to *cis* isomers and lignin (1644, 1660 cm^−1^) [45], polygalacturonic (pectic) acid from 1249 cm^−1^, and carbohydrates from the bands positioned at 1407, 1426, 472, and 486 cm^−1^ [34] (Figure 8A–C).

According to the score plot based on the FTIR spectra of tomatoes (Figure 8D,E), PC2 indicated clear differences between CS and other varieties according to the higher-intensity bands indicated on fatty acids (1739 cm^−1^), cellulose (1737, 1759, 1624, 1510, 1473 cm^−1^), hemicellulose (1668 cm^−1^), and lignin (1174 cm^−1^), while bands at 2860–2847 and ~2920–2940 were asymmetric and symmetric stretching vibrations of the CH groups, which could be associated with the hydrocarbon chains of the carbohydrates or lignin [33,34,35,36,38]. On the other hand, several bands (Figure 5F) were attributed to fatty acids, including the carbonyl C=O stretching band at 1743 cm^−1^ and the CH_2_ scissoring vibrations at 1454 cm^−1^ [38].

Since there is no information about components (bands) that could be responsible for the separation of the investigated samples, a pattern-based approach was applied. This approach allows the simultaneous recording of a number of compounds, and, together with chemometrics (pattern recognition), it could provide respectable information about the biological sample. When it comes to preprocessing algorithms, it is not possible to determine a priori which approach should be applied. Therefore, several approaches were investigated in order to find the one that provided the best classification power.

Hence, the best accuracy performance was obtained using Raman spectroscopy with the SVM algorithm: 100.00, 96.25, and 92.71% for lettuce, tomato, and paprika varieties, respectively. Further, FT-IR spectroscopy displayed the best discrimination accuracy in combination with SVM for lettuce (99.37%), tomato (97.50%), and paprika (92.50%) varieties (Table 1, Table 2 and Table 3).

The results obtained using Raman spectroscopy in combination with PCA-QDA showed the best accuracy when MSC was used. The discrimination results were 100.00, 60.50, and 100.00% for lettuce, paprika, and tomato varieties, respectively (Table 1, Table 2 and Table 3).

PLS-DA showed the worst performance in terms of classification using both methods: Raman and FT-IR spectroscopy. In the case of Raman spectroscopy, PLS-DA was not suitable for the classification of the studied lettuce genotypes (0%), while, for paprika and tomato accuracy, it ranged from 30.26 to 47.36% and from 1.31 to 84.21%, respectively. On the other hand, the results of the FT-IR spectroscopy ranged from 10.53 to 19.74, 14.56 to 50.00, and 5.26 to 57.89% for lettuce, paprika, and tomato varieties, respectively, depending on the preprocessing approach (Table 1, Table 2 and Table 3).

### 2.4. Merging Raman and FT-IR Spectral Information

**Figure 9:**
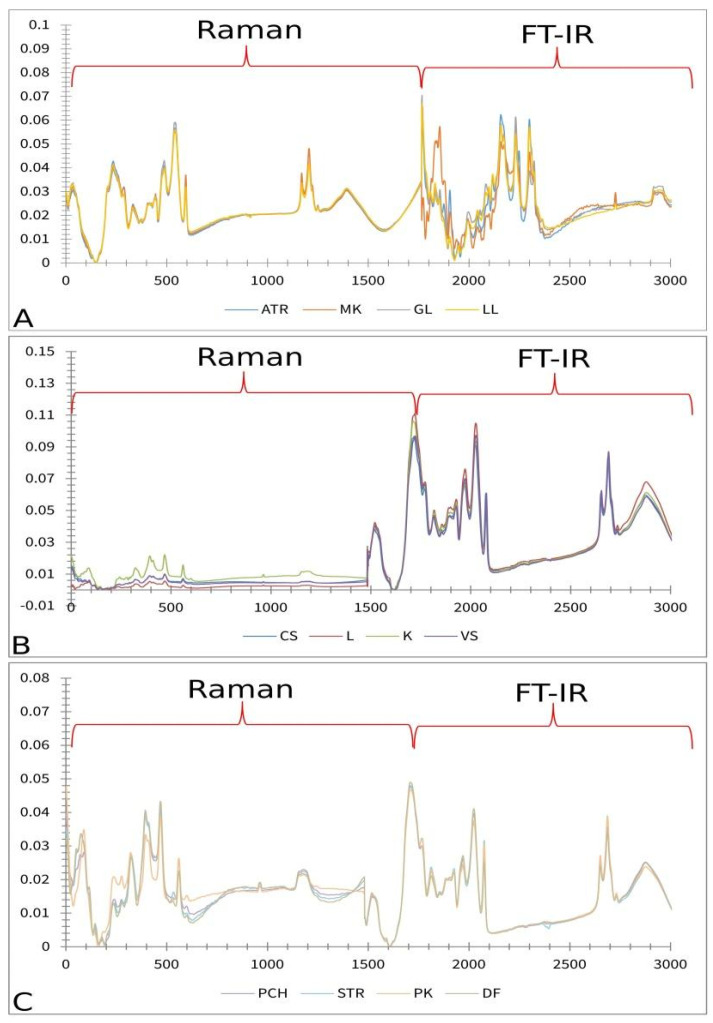
Preprocessed and merged Raman and FT-IR spectra; (**A**) lettuce: ATR—Atrakcija, MK—Majska kraljica, GL—Great Lake, LL—Ljubljanska ledenka; (**B**) tomato: CS—Crvena Stena, L—Lider F1, K—King F1, VS—Volovsko srce; (**C**) paprika: PCH—Palanačko čudo, STR—Strižanka, PK—Palanačka kapija, DF—Delfina.

**Figure 10:**
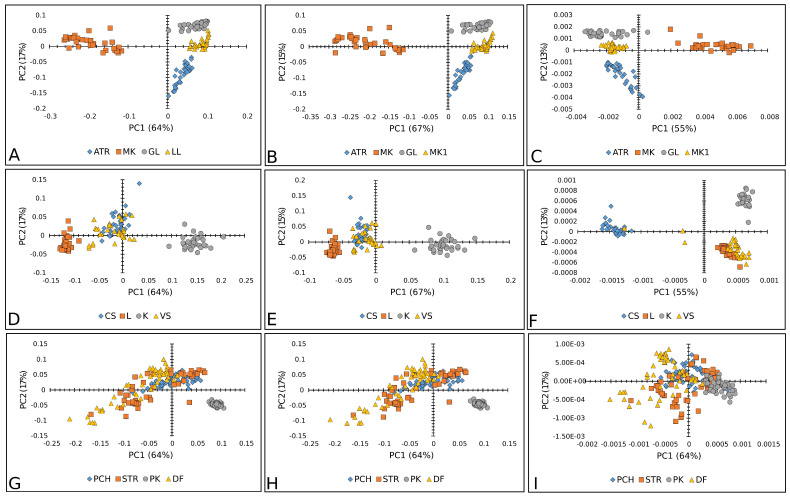
PCA score plots (PC1 vs. PC2) of lettuce varieties: Raman+FT-IR spectra combined with BC+N (**A**), BC+N+MSC (**B**), and BC+N+MSC+2nd OD (**C**); tomato varieties: Raman+FT-IR spectra with BC+N (**D**), BC+N+MSC (**E**), and BC+N+MSC+2nd OD (**F**); paprika varieties: Raman+FT-IR spectra with BC+N (**G**), BC+N+MSC (**H**), and BC+N+MSC+2nd OD (**I**). ATR—Atrakcija, MK—Majska kraljica, GL—Great Lake, LL—Ljubljanska ledenka; CS—Crvena Stena, L—Lider F1, K—King F1, VS—Volovsko srce; PCH—Palanačko čudo, STR—Strižanka, PK—Palanačka kapija, DF—Delfina.

In order to improve the classification accuracy, the data obtained by Raman and FT-IR spectroscopy were merged. Firstly, the FT-IR spectra of each species and each variety were transformed from transmittance to absorbance and then normalized, and the baseline was corrected. Raman spectra were baseline-corrected and normalized, and then the information was put in the same matrix and plotted (Figure 9). Preprocessing was performed in the same way as in the case of non-merged Raman and FT-IR data. Preprocessed data underwent data redaction by PCA. Score plots clearly showed the differences in the spectra of different varieties within all of the three selected vegetable crops (Figure 10). Scores were further used as input for classification analysis in the same manner as in the previous analysis.

**Figure 11:**
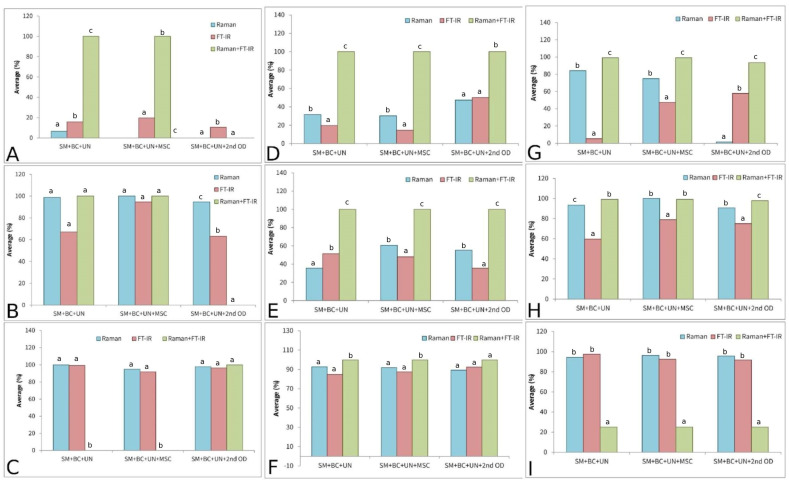
Results of average classification accuracy performed on lettuce seed varieties: PLS-DA (**A**), PCA-QDA (**B**), SVM (**C**); paprika seed varieties: PLS-DA (**D**), PCA-QDA (**E**), SVM (**F**); tomato seed varieties: PLS-DA (**G**), PCA-QDA (**H**), SVM (**I**). Different small letters at each figure (**A**–**I**) implies statistical significant differences (*p* < 0.05).

It is clearly displayed that merging the data obtained using two vibrational spectroscopy tools demonstrated better prediction performance compared to a single spectroscopy approach. In the case of the lettuce varieties, the merged spectra contributed to better accuracy in terms of the classification system. Accordingly, all three applied models (PLS-DA, PCA-QDA, and SVM) reached 100% accuracy in terms of the merged spectra (Table 1 and Figure 11). The highest improvement was noted for the PLS-DA model, indicating that two-class models could operate as multiclass models when a sufficient amount of information is available. In the case of paprika seed varieties, the results showed an even more drastic difference when compared to models based on information obtained separately from the Raman and FT-IR instruments. The improvement in accuracy was 100.00% on average for all the models used in all the preprocessing approaches (Figure 11 and Table 2). In the case of the tomato varieties, the maximal classification accuracy (100%) was obtained, with an exception for the SVM model (Figure 11 and Table 3). Generally, models developed using information obtained from two analytical techniques tended to perform with better accuracy. This was expected, as incorporating more chemical information during model development enhances the model’s performance.

## 3. Discussion

Generally, PLS-DA is a linear method that is suitable for two-class separation rather than multi-class separation, which may be the main reason for its weak discrimination power. However, QDA provides a non-linear quadratic decision boundary, which provides better separation performance. An SVM is basically a linear method, but, in this research, a polynomial kernel was used. In machine learning, the polynomial kernel is a kernel function commonly used with the SVM and other kernelized models, which represents the similarity of vectors (training samples) in a feature space over polynomials of the original variables, allowing learning of non-linear models [42]. In general, the kernel allows SVMs to handle nonlinearly separable datasets. Most likely, that is why the QDA and SVM showed the best accuracy results.

The results of classification according to the results obtained by Raman spectroscopy displayed an accuracy of around 93% for all species when SVM was used. Similar results were obtained in the case of FT-IR spectroscopy, where over 92% of samples were properly classified using the SVM algorithm. A combination of Raman and FT-IR spectroscopy techniques improved the classification accuracy, where the results for lettuce, tomato, and paprika were 100% (SVM, PCA-QDA, PCA-LDA), 100% (SVM), and 95% (PCA-QDA), respectively.

Generally, models developed using information obtained from two analytical techniques tend to perform with better accuracy. This is expected, as incorporating more chemical information during model development enhances the model’s performance. The accuracy of the classification was improved due to more detailed and more informative inputs. It seems that merging Raman and FT-IR spectra enhances classification power and model reliability by leveraging the complementary strengths of both spectroscopy techniques operating in different spectral modes. Raman spectroscopy is sensitive to changes in molecular polarizability, making it particularly effective for detecting symmetric stretches and nonpolar bonds [43], while FT-IR spectroscopy focuses on changes in the dipole moment, highlighting polar functional groups and asymmetric vibrations [46]. By combining these spectra, a more comprehensive and detailed feature space is created, capturing a broader range of molecular vibrations and subtle chemical differences. Such an enhanced dataset improves the signal-to-noise ratio, increases sensitivity and specificity, and enables more accurate and reliable discrimination between similar compounds or complex mixtures. Consequently, classification models built on merged spectra are better equipped to detect subtle differences, thus providing more robust and precise predictions. Therefore, they could be applied in studies of different composite materials, such as foods and other biological samples.

Since the chemical composition of seeds depends on both agroecological factors (such as climate, soil characteristics, and water availability) and genetic factors, the chemical structure of seed material can vary from year to year [47]. Therefore, repeating experiments and incorporating artificial intelligence (AI) to learn from new data should be considered as a future direction.

## 4. Materials and Methods

### 4.1. Seed Material

**Figure 12:**
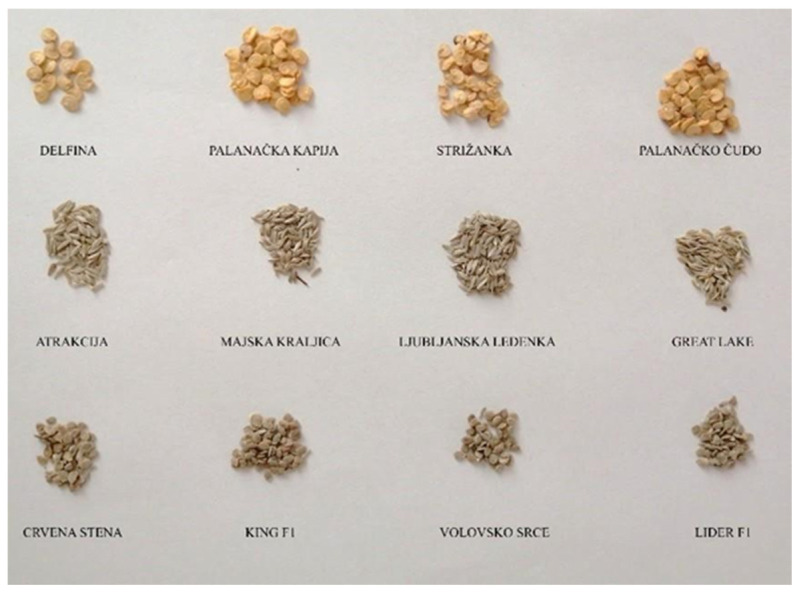
Seed material of different varieties (first row-paprika; second row-lettuce; third row-tomato).

Seed material was obtained from certified seed producers (Superior, Velika Plana; Institut of Field and Vegetable Crop, Novi Sad; Institute for Vegetable Crops, Smederevska Palanka). In total, 12 varieties of tested crops were analyzed (4 varieties of tomato, 4 of paprika, and 4 of lettuce). From each variety, 50 seeds were taken and milled (each seed separately) (BioSpec Products, Inc., Bartlesville, OK, USA) because of the high fluorescence produced by the seed coat. Milled seeds were subjected to Raman and FT-IR spectroscopy recording. Figure 12 presents investigated seed samples.

### 4.2. FT-IR Spectroscopymeasurements

Fourier transform infrared spectroscopy (FT-IR) analysis was performed on the ground seed samples using an IRAffinity-1 FT-IR spectrophotometer (Schimadzu, Kyoto, Japan). The wavelength range was 4000–600 cm^−1^, with 256 interferometer scans added for each spectrum, while the resolution was 4 cm^−1^. In total, 50 spectra were recorded per variety (one milled seed, one spectrum).

### 4.3. Raman Spectroscopy Acquisition

Raman microspectroscopy was carried out using an XploRA Raman spectrometer (Horiba Jobin Yvon, Kyoto, Japan). Raman scattering was carried out using a laser at a wavelength of 785 nm (with a maximum output power of 20–25 mW) equipped with a 600-line mm^−1^ grating. The measurement was conducted with a 5 s exposure time and 5 spectral accumulations. The samples were scanned in the common range of 200 to 3400 cm^−1^ with a 3 cm^−1^ spectral resolution. Autocalibration was carried out daily prior to the recording of spectra using a 520.47 cm^−1^ line of silicon. In total, 50 spectra were recorded per variety (one milled seed, one spectrum).

### 4.4. Chemometric Analysis

The raw spectra obtained by Raman and FT-IR instrumentation were arranged in matrices with 200 rows and 1481 columns and 200 rows and 1764 columns, respectively. Spectra were divided into a training set (70% of total spectra) and a test set (30%). The training set was used to train the model to find patterns and relationships within the data, while the test set was used to test the model’s performance based on new (unseen) data. Because of the difficulty in choosing a proper preprocessing method that would be suitable for all mathematical classification models and data, it was necessary to check several preprocessing techniques. In this paper, smoothing (SM), linear baseline correction (BC), unit vector normalization (UN), multiplicative scatter correction (MSC), and second-order derivative (2nd OD) were chosen in the following arrangement: (1) smoothing + linear baseline correction + unit vector normalization; (2) smoothing + linear baseline correction + unit vector normalization + full multiplicative scatter correction (MSC); (3) smoothing + baseline correction + unit vector normalization + second-order derivative. After pre-processing work, principal component analysis (PCA) was applied. According to the eigenvalue, four PCs were included for further analysis. The obtained PCs served as an input for the Support Vector Machines (SVM) algorithm, Partial Least Square Discriminant Analysis (PLS-DA), and Principal Component Analysis-Quadratic Discriminant Analysis (PCA-QDA).

A support vector machine (SVM) is a supervised computer algorithm that assigns labels to objects by learning from examples [48]. The essence of an SVM lies in four basic concepts: the separating hyperplane, the maximum-margin hyperplane, the soft margin, and the kernel function [49]. A separating hyperplane simply represents the line that separates all data points [50].

PLS-DA is based on a PLS regression algorithm, which looks for latent variables (LVs) with the highest covariance with the Y-variables. PLS-DA aims to create models that optimize the separation between classes of objects [51].

A QDA classifier assumes that each class has its own covariance and produces a quadratic boundary. The QDA optimally discriminates between the classes in the dataset and requires large computation and data [52].

### 4.5. Preprocessing

The aim of the smoothing procedure is to eliminate noises that corrupt Raman spectra and can be applied before (as was the case in our study) or after baseline correction. There are a lot of smoothing procedures, such as the Savitzky-Golay method, Gaussian, median filtering, and mean. In our experiment, the Savitzky-Golay method was performed because it is the most effective in preserving the bands from corruption [53].

Baseline correction represents an important part of Raman spectroscopy-based chemometric analysis because of its ability to extract the true Raman band intensities necessary for further numerical processing [54]. If baseline correction is not properly conducted, the resulting artifacts may obstruct further data analysis, such as classification.

In some cases, there are variations in the intensity between samples. The reason for this lies in the alteration of some experimental factors and focusing during the measurement of spectra. These problems could be overcome by performing unit vector (SNV). SNV is based on two subsequent steps: subtracting each spectrum by its own mean and then dividing it by its own standard deviation [55]. Multiplicative scatter correction (MSC) aims to correct spectra for grouping closely to a reference spectrum by changing the scale and the offset of the spectra.

The second derivative of the spectra is suitable for the evaluation of the band position. In comparison to the original bands (or the whole spectra), the full width at half maximum (FWHM) is smaller for the band in the second derivative. As a result, two bands can be distinguished in the second derivative, although they are not recognized in the original signal [56].

The results of the classification analysis are represented as an average accuracy (in %) based on the results obtained by the test set of the data for each variety. It is defined as a total number of correctly classified samples (in the test set) for each variety divided by the total number of samples for each variety (in the test set) × 100. Average classification accuracy is defined as the sum of the total number of corrected classified samples for all varieties divided by the total number of samples × 100. All chemometrics analyses (preprocessing and modeling) were performed using Unscrambler 10.4 (CAMO software, Oslo, Norway). The comparison of the results obtained by different instrumental approaches (Raman, FT-IR, and Raman+FT-IR) was conducted using analysis of variance. Tukey’s post hoc tests were carried out to assess the significant differences indicated by the ANOVA results (*p* < 0.05). ANOVA and post hoc tests were performed using SPSS 26 (SPSS Inc., Chicago, IL, USA).

## 5. Conclusions

In this study, Raman and FT-IR spectroscopy were coupled with chemometrics to develop an appropriate model for the classification of different varieties’ seeds of lettuce, paprika, and tomato species, i.e., within the target vegetable species as a consequence of variability in the studied crop varieties.

The results showed that in the case of Raman spectroscopy, the best results were obtained using SVM in combination with baseline correction and unit vector normalization for all varieties of the three studied vegetable species. The same results were obtained for FT-IR, except for paprika, where the best classification accuracy was displayed by SVM in combination with smoothing, baseline correction, and a second-order derivative. Generally, models made according to Raman spectra showed better classification power in comparison with FT-IR spectra, probably because there are more differences (variations) in molecules that are sensitive to Raman spectroscopy. This is probably because Raman gives more information about samples and, consequently, more differences (variations) in Raman spectra between varieties compared to FT-IR spectra.

Our results pointed out the prospect for the future use of non-targeted, fast, non-preparatory, and low-cost spectroscopy techniques, such as Raman and FT-IR, in the discrimination of seeds of different varieties. Therefore, as an alternative approach to varietal identification and genetic purity testing, vibrational spectroscopy can contribute to a better quality of seed material, prevent market frauds, and, consequently, affect the quality of raw material for the food industry. A novel approach of merging Raman and FT-IR spectra resulted in improved and more reliable classification results. To improve the reliability of these models and facilitate their practical application, future investigations should be focused on repeating the experiment and conducting 2D and 3D Raman imaging. This will allow for the acquisition of more comprehensive chemical information, particularly in terms of the distribution of specific components. The reliability of these models can also be bolstered by integrating artificial intelligence (AI), allowing the models to learn from new datasets. Ensuring the repeatability of these models over time will probably provide a reliable and rapid tool for possibly evaluating plant genetic resources. The problem of overfitting and unreliable inference beyond the studied samples may reduce confidence in this approach when these models are applied to large genotype collections.

## Figures and Tables

**Table 1:** Results of classification accuracy of different lettuce genotypes analyzed by Raman and FT-IR spectroscopy.

Type of Spectroscopy	Type of Models	Type of Preprocessing	Varieties				Average (%)
			Atrakcija	Majska Kraljica	Great Lakes	Ljubljanska Ledenka	
			Test (%)	Test (%)	Test (%)	Test (%)	
Raman	PLS-DA	SM+BC+UN	0.00	0.00	26.32	0.00	6.58
		SM+BC+UN+MSC	0.00	0.00	0.00	0.00	0.00
		SM+BC+UN+2nd OD	0.00	0.00	0.00	0.00	0.00
	PCA-QDA	SM+BC+UN	100	100	94.74	100	98.69
		SM+BC+UN+MSC	100	100	100	100	100.00
		SM+BC+UN+2nd OD	100	100	78.95	100	94.74
	SVM	SM+BC+UN	100.00	100.00	100.00	100.00	100.00
		SM+BC+UN+MSC	95.00	95.00	95.00	95.00	95.00
		SM+BC+UN+2nd OD	98.00	98.00	98.00	98.00	98.00
FT-IR	PLS-DA	SM+BC+UN	31.58	15.79	0.00	15.79	15.79
		SM+BC+UN+MSC	63.18	15.79	0.00	0.00	19.74
		SM+BC+UN+2nd OD	15.79	0.00	0.00	26.32	10.53
	PCA-QDA	SM+BC+UN	10.53	63.18	100.00	94.74	67.11
		SM+BC+UN+MSC	84.21	94.74	100.00	100.00	94.74
		SM+BC+UN+2nd OD	57.89	89.47	68.42	36.84	63.15
	SVM	SM+BC+UN	97.50	100.00	100.00	100.00	99.37
		SM+BC+UN+MSC	97.50	90.00	90.00	90.00	91.87
		SM+BC+UN+2nd OD	92.50	93.33	100.00	100.00	96.46
Raman + FT-IR	SVM	SM+BC+UN	100.00	0.00	0.00	0.00	25.00
		SM+BC+UN+MSC	100.00	0.00	0.00	0.00	25.00
		SM+BC+UN+2nd OD	100.00	100.00	100.00	100.00	100.00
	PCA-QDA	SM+BC+UN	100.00	100.00	100.00	100.00	100.00
		SM+BC+UN+MSC	100.00	100.00	100.00	100.00	100.00
		SM+BC+UN+2nd OD	100.00	0.00	0.00	0.00	25.00
	PCA-LDA	SM+BC+UN	100.00	100.00	100.00	100.00	100.00
		SM+BC+UN+MSC	100.00	100.00	100.00	100.00	100.00
		SM+BC+UN+2nd OD	100.00	0.00	0.00	0.00	25.00

**Table 2:** Results of classification accuracy of different paprika genotypes analyzed by Raman and FT-IR spectroscopy.

Type of Spectroscopy	Type of Models	Type of Preprocessing	Varieties				Average (%)
			Palanacko Čudo	Strizanka	Palanačka Kapija	Delfina	
			Test (%)	Test (%)	Test (%)	Test (%)	
Raman	PLS-DA	SM+BC+UN	31.58	0.00	57.90	36.84	31.58
		SM+BC+UN+MSC	26.32	0.00	57.90	36.84	30.26
		SM+BC+UN+2nd OD	68.42	42.10	78.94	0.00	47.36
	PCA-QDA	SM+BC+UN	89.47	0.00	5.26	47.37	35.52
		SM+BC+UN+MSC	89.47	42.10	5.26	47.37	60.50
		SM+BC+UN+2nd OD	47.37	21.05	63.16	89.47	55.26
	SVM	SM+BC+UN	83.00	83.33	100.00	100.00	92.71
		SM+BC+UN+MSC	87.50	80.00	100.00	100.00	91.87
		SM+BC+UN+2nd OD	97.50	80.00	90.00	90.00	89.37
FT-IR	PLS-DA	SM+BC+UN	10.53	42.10	21.05	5.26	19.73
		SM+BC+UN+MSC	16.13	0.00	0.00	42.12	14.56
		SM+BC+UN+2nd OD	42.10	57.89	0.00	100.00	50.00
	PCA-QDA	SM+BC+UN	73.68	21.05	78.95	31.58	51.31
		SM+BC+UN+MSC	42.10	36.82	12.90	100.00	47.95
		SM+BC+UN+2nd OD	19.35	6.45	16.13	100.00	35.48
	SVM	SM+BC+UN	82.50	86.67	85.00	85.00	84.79
		SM+BC+UN+MSC	85.00	85.00	90.00	90.00	87.50
		SM+BC+UN+2nd OD	90.00	90.00	95.00	95.00	92.50
Raman + FT-IR	SVM	SM+BC+UN	79.00	79.00	100.00	79.00	84.25
		SM+BC+UN+MSC	79.00	79.00	100.00	79.00	84.25
		SM+BC+UN+2nd OD	95.00	79.00	100.00	79.00	88.25
	PCA-QDA	SM+BC+UN	90.00	86.00	100.00	84.00	90.50
		SM+BC+UN+MSC	88.00	84.00	100.00	86.00	89.50
		SM+BC+UN+2nd OD	100.00	88.00	98.00	94.00	95.00
	PCA-LDA	SM+BC+UN	100.00	0.00	0.00	0.00	25.00
		SM+BC+UN+MSC	100.00	0.00	0.00	0.00	25.00
		SM+BC+UN+2nd OD	100.00	0.00	0.00	0.00	25.00

**Table 3:** Results of classification accuracy of different tomato genotypes analyzed by Raman and FT-IR spectroscopy.

Type of Spectroscopy	Type of Models	Type of Preprocessing	Varieties				Average (%)
			Crvena Stena	King F1	Volovsko Srce	Lider F1	
			Test (%)	Test (%)	Test (%)	Test (%)	
Raman	PLS-DA	SM+BC+UN	63.16	100.00	100.00	73.68	84.21
		SM+BC+UN+MSC	52.63	100.00	100.00	47.37	75.00
		SM+BC+UN+2nd OD	0.00	0.00	0.00	5.26	1.31
	PCA-QDA	SM+BC+UN	89.47	94.74	100.00	89.47	93.42
		SM+BC+UN+MSC	100.00	100.00	100.00	100.00	100.00
		SM+BC+UN+2nd OD	89.47	89.47	100.00	84.21	90.79
	SVM	SM+BC+UN	92.50	100.00	85.00	100.00	94.38
		SM+BC+UN+MSC	85.00	100.00	100.00	100.00	96.25
		SM+BC+UN+2nd OD	82.50	100.00	100.00	100.00	95.62
FT-IR	PLS-DA	SM+BC+UN	0.00	15.79	0.00	5.26	5.26
		SM+BC+UN+MSC	100.00	63.16	0.00	26.32	47.37
		SM+BC+UN+2nd OD	89.47	36.84	52.63	52.63	57.89
	PCA-QDA	SM+BC+UN	89.47	42.10	47.37	100.00	59.65
		SM+BC+UN+MSC	100.00	84.21	42.10	89.47	78.94
		SM+BC+UN+2nd OD	89.47	73.68	47.37	89.47	75.00
	SVM	SM+BC+UN	90.00	100.00	100.00	100.00	97.50
		SM+BC+UN+MSC	80.00	100.00	95.00	95.00	92.50
		SM+BC+UN+2nd OD	77.50	100.00	95.00	95.00	91.87
Raman + FT-IR	SVM	SM+BC+UN	100.00	100.00	100.00	100.00	100.00
		SM+BC+UN+MSC	100.00	100.00	100.00	100.00	100.00
		SM+BC+UN+2nd OD	100.00	88.89	100.00	86.11	93.55
	PCA-QDA	SM+BC+UN	100.00	100.00	100.00	97.22	99.19
		SM+BC+UN+MSC	100.00	100.00	100.00	97.22	99.19
		SM+BC+UN+2nd OD	100.00	100.00	100.00	91.67	97.92
	PCA-LDA	SM+BC+UN	100.00	0.00	0.00	0.00	25.00
		SM+BC+UN+MSC	100.00	0.00	0.00	0.00	25.00
		SM+BC+UN+2nd OD	100.00	0.00	0.00	0.00	25.00

## Data Availability

The original contributions presented in this study are included in the article. Further inquiries can be directed to the corresponding author(s).
